# "WHAT'S YOURS IS MINE": A DYADIC STUDY OF EVERYDAY EMOTIONAL EXPERIENCES AND CORTISOL IN OLDER COUPLES

**DOI:** 10.1093/geroni/igac059.731

**Published:** 2022-12-20

**Authors:** Theresa Pauly, Karolina Kolodziejczak, Johanna Drewelies, Maureen Ashe, Kenneth Madden, Nilam Ram, Denis Gerstorf, Christiane Hoppmann

**Affiliations:** University of Zurich, Zurich, Zurich, Switzerland; Medical School Berlin, Berlin, Berlin, Germany; Humboldt University Berlin, Berlin, Berlin, Germany; The University of British Columbia, Vancouver, British Columbia, Canada; University of British Columbia, Vancouver, British Columbia, Canada; Stanford University, Stanford, California, United States; Humboldt Universität zu Berlin, Berlin, Berlin, Germany; University of British Columbia, Vancouver, British Columbia, Canada

## Abstract

Older partners’ health is linked; individuals’ physiological arousal can be shaped by their own and their partner’s emotional experiences. This study examines those associations using repeated daily life assessments (7 days) of physiological arousal (cortisol), positive affect (PA), and negative affect (NA) obtained from 321 older couples. Results from multi-level models revealed, at the between-person level, that individuals with higher average cortisol had partners with higher overall NA; and, at the within-person (momentary) level, that cortisol was lower in moments when the partner’s PA was higher than usual and when one’s own PA was higher and NA was lower than usual. On a day level, cortisol output was lower on days with lower own NA and with higher partner PA, particularly when relationship satisfaction was high, but unrelated to own PA. The findings emphasize the importance of considering how members of a dyad influence each other’s health in old age.

